# SARS-COV-ATE risk assessment model for arterial thromboembolism in COVID-19

**DOI:** 10.1038/s41598-022-18510-3

**Published:** 2022-09-28

**Authors:** Pin Li, Yi Lee, Qasim Jehangir, Chun-Hui Lin, Geetha Krishnamoorthy, Anupam A. Sule, Abdul R. Halabi, Kiritkumar Patel, Laila Poisson, Girish B. Nair

**Affiliations:** 1 Department of Public Health Sciences, Henry Ford Health, Detroit, MI USA; 2 Department of Medicine, Trinity Health St. Joseph Mercy Oakland Hospital, Pontiac, Ml USA; 3 Wayne State University School of Medicine, Detroit, MI USA; 4 Division of Cardiology, Trinity Health St. Joseph Mercy Oakland Hospital, Pontiac, MI USA; 5 Department of Informatics, Trinity Health St. Joseph Mercy Oakland Hospital, Pontiac, MI USA; 6 Oakland University William Beaumont School of Medicine, Auburn Hills, MI USA; 7 Division of Pulmonary and Critical Care Medicine, William Beaumont Hospital, Royal Oak, MI USA

**Keywords:** Risk factors, Myocardial infarction

## Abstract

Patients with SARS-CoV-2 infection are at an increased risk of cardiovascular and thrombotic complications conferring an extremely poor prognosis. COVID-19 infection is known to be an independent risk factor for acute ischemic stroke and myocardial infarction (MI). We developed a risk assessment model (RAM) to stratify hospitalized COVID-19 patients for arterial thromboembolism (ATE). This multicenter, retrospective study included adult COVID-19 patients admitted between 3/1/2020 and 9/5/2021. Among 3531 patients from the training cohort, 15.5% developed acute in-hospital ATE, including stroke, MI, and other ATE, compared to 13.4% in the validation cohort. The 16-item final score was named SARS-COV-ATE (Sex: male = 1, Age [40–59 = 2, > 60 = 4], Race: non-African American = 1, Smoking = 1 and Systolic blood pressure elevation = 1, Creatinine elevation = 1; Over the range: leukocytes/lactate dehydrogenase/interleukin-6, B-type natriuretic peptide = 1, Vascular disease (cardiovascular/cerebrovascular = 1), Aspartate aminotransferase = 1, Troponin-I [> 0.04 ng/mL = 1, troponin-I > 0.09 ng/mL = 3], Electrolytes derangement [magnesium/potassium = 1]). RAM had a good discrimination (training AUC 0.777, 0.756–0.797; validation AUC 0.766, 0.741–0.790). The validation cohort was stratified as low-risk (score 0–8), intermediate-risk (score 9–13), and high-risk groups (score ≥ 14), with the incidence of ATE 2.4%, 12.8%, and 33.8%, respectively. Our novel prediction model based on 16 standardized, commonly available parameters showed good performance in identifying COVID-19 patients at risk for ATE on admission.

## Introduction

Patients with SARS-CoV-2 infection are at an increased risk of cardiovascular and thrombotic complications portending an extremely poor prognosis^1,2^. These complications range from acute ischemic stroke, myocardial infarction (MI), cardiomyopathy, intracardiac thrombus, dysrhythmias, to thromboembolism^1–3^. Cardiovascular complications are not limited to COVID-19 infection and are also seen in other viral illnesses, including influenza^4,5^ and severe acute respiratory syndrome^6,7^. The venous thromboembolic complications are more frequent in COVID-19 compared to influenza^8,9^ whereas data on the incidence of arterial complications is conflicting^8,10,11^. The incidence of stroke and dysrhythmias is higher in COVID-19 compared to influenza^3,10^ while myocardial injury is reportedly more common in influenza patients^11^.

COVID-19 infection is known to be an independent risk factor for stroke and MI^12^; however, data on the risk factors of arterial thromboembolism (ATE) in COVID-19 infection is limited. Early risk stratification in COVID-19 infection is crucial as it can identify the patients at high risk for experiencing ATE events during the hospital course. These patients might benefit from intervention strategies, including optimization of prophylactic antiplatelet and antithrombotic therapy, closer monitoring through serial clinical exams, and wider provision of diagnostic resources. This, in turn, can potentially lead to improved patient outcomes.

There is a need for a model which can assist clinicians in the emergency room in predicting the risk of ATE in COVID-19 patients. This model should be easily applicable in clinical practice, uses readily available parameters, and does not require detailed calculations. Risk assessment models (RAM) have been proposed for venous thromboembolism, including 3D-PAST for hospitalized COVID-19 patients^13^ and CoVID-TE for COVID-19 patients with cancer^14^; however, similar models are not available for ATE. In this study, we sought the incidence of cardiovascular, cerebrovascular, and other ATE complications in a large cohort of hospitalized COVID-19 patients. We comprehensively assessed the potential risk factors and common biomarkers, leading to a robust, integer-based RAM. The model was further validated by bootstrapping for reproducibility and validated by a subsequent population from the same clinical setting for generalizability.

## Method

### Study setting

This is a retrospective, multi-institutional (one quaternary care and three community hospitals) cohort study of patients older than 18 years old admitted with polymerase chain reaction proven SARS-CoV-2 infection between March 1, 2020, and September 5, 2021. Data were analyzed from the Southeast Michigan COVID-19 Consortium Registry Database, a multi-institutional database of four main health systems in southeast Michigan, United States, including Henry Ford Health System, Beaumont Health System, Trinity Health System, and Wayne State University^15,16^. Data from Trinity and Henry Ford Health were used for this particular study. This study was approved by the Trinity Health institutional review board, which waived the need for informed consent for the use of de-identified medical records. All methods were performed in accordance with the Declaration of Helsinki. The collected data included baseline demographics, past medical history, presenting vital signs, and initial laboratory values for all adult patients.

### Risk assessment model development

The model was built and tested using the previously validated method^13^. The primary composite outcome was acute ATE events, including acute ischemic stroke, transient ischemic attack, MI, unstable angina, intracardiac thrombus, mesenteric ischemia, peripheral thromboembolism, and other ATE as identified by standard-text variables and International Classification of Diseases–Tenth Revision codes (Supplemental Table 1). The patients admitted between March 1, 2020, and December 31, 2020, were considered as the training cohort. Multivariate imputation by chained equations (MICE) was conducted to impute missing values for variables with missingness. An imputed dataset was derived by using predictive mean matching for numeric variables, logistic regression for binary variables, and Bayesian polytomous regression for factor variables. Initial descriptive statistics were reported, the mean and standard deviation for continuous variables and proportions for categorical variables. All continuous variables were categorized (Supplemental Table 2). For group comparison by arterial events, the t-test was conducted for continuous variables and the Chi-square test for categorical variables. With relatively small event numbers compared to the abundant variables included in our study, Least Absolute Shrinkage and Selection Operator (LASSO) regression was applied to minimize potential collinearity and over-fitting. Multivariable logistic regression was then conducted to assess the relationship between arterial events and selected factors. Integer scores were assigned based on the estimated coefficients, and the total score for each patient was calculated. The receiver operating characteristic curve (ROC) and area under the curve (AUC) were used to evaluate the performance of the total score. Cut-off points for low, moderate, and high risk of ATE were determined based on the total score and were used to stratify the patients.

### Risk assessment model validation

Based on training data, bootstrapping validation was done on a bootstrapped cohort (*N* = 500). An independent data set of patients admitted between January 1, 2021, and September 5, 2021, was used as external validation. The same inclusion criteria and data cleaning process was applied to the validation cohort as the training cohort. The performance, including ROC, AUC, sensitivity, specificity, positive and negative predictive values, was calculated and compared between the training cohort, bootstrapped cohort, as well as validation cohort.

All statistical tests were 2-sided with an α (significance) level of 0.05. All data were analyzed using R version 4.0.4^17^.

## Results

### Patient characteristics

The model was created using a derivation population of 3526 patients (baseline characteristics are shown in Supplemental Table 3A. In comparison to patients with no ATE, ATE patients were significantly older and more often males and Caucasians. Patients with ATE were also more hypoxic on arrival. Moreover, the prevalence of comorbidities such as hypertension, hyperlipidemia, coronary artery disease (CAD), congestive heart failure, cerebrovascular accident (CVA), atrial fibrillation, and chronic kidney disease was higher in ATE patients. There were 599 ATE events among 547 patients: 418 patients had MI, 44 had stroke, 39 had transient ischemic attack, 7 had intracardiac thrombus, 2 had vascular disorders of intestine, 1 had unstable angina, 88 had other ATE (Supplemental Table 4). A total of 50 patients had 2 ATE events, and 1 patient had 3 ATE events.

### Risk score development

**Figure 1:**
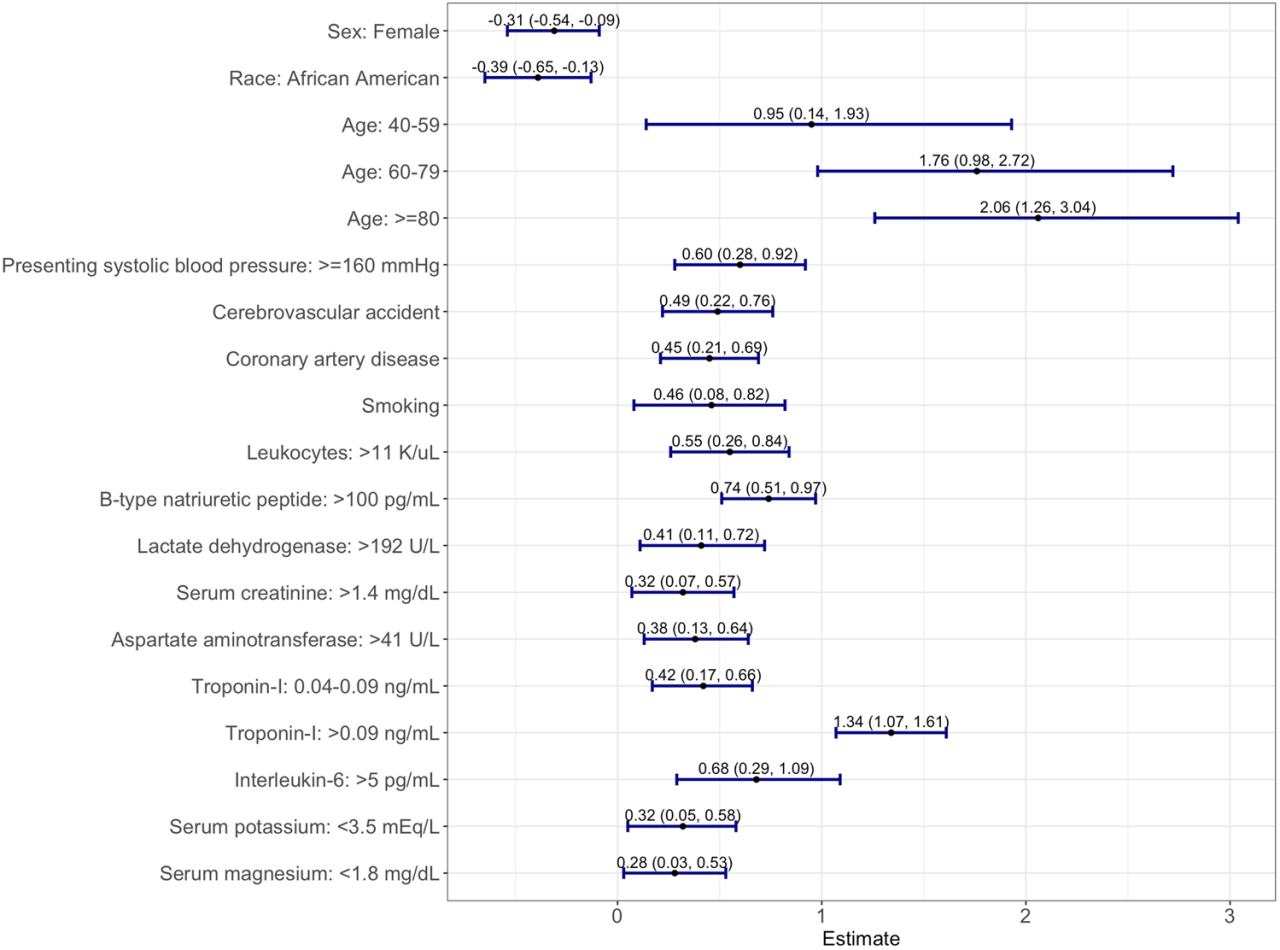
Multivariable analysis of the factors associated with risk of arterial thromboembolism in patients hospitalized with COVID-19.

**Figure 2:**
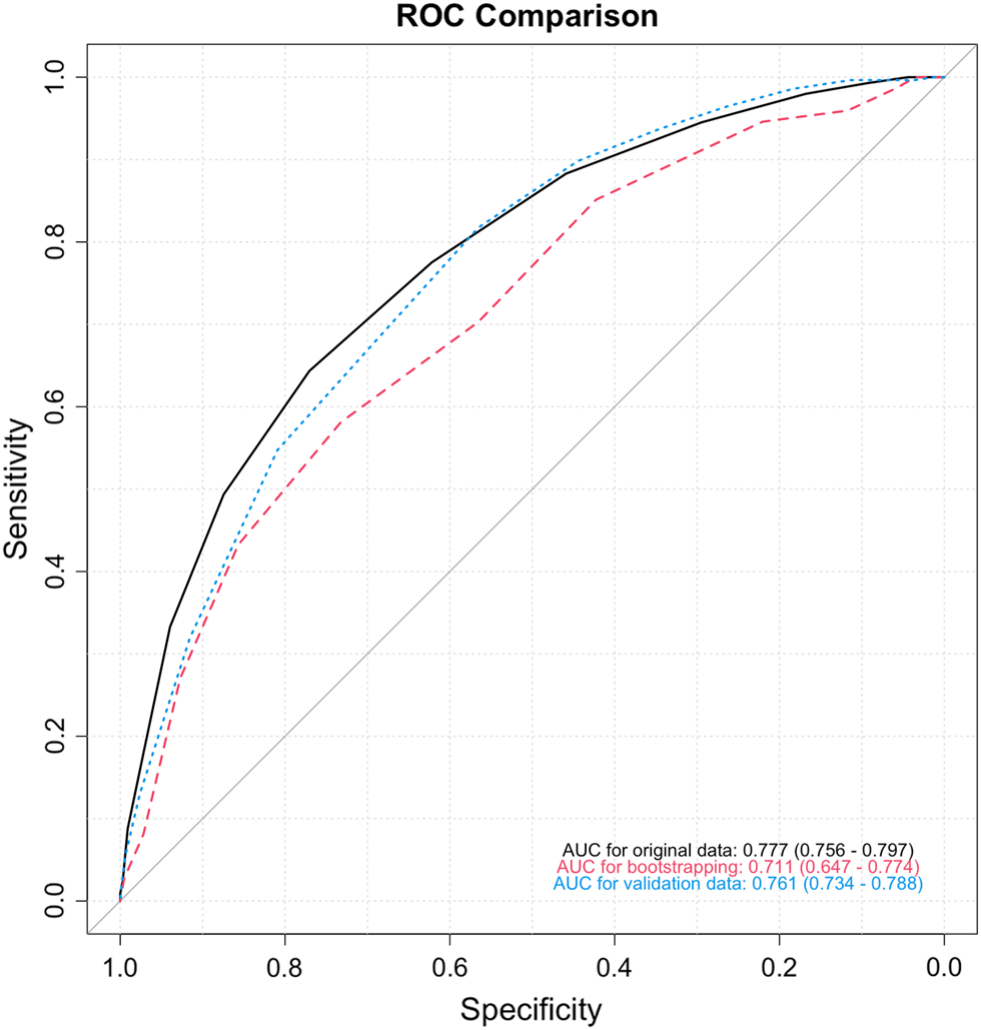
Receiver operating characteristic curves for derivation cohort, bootstrapped cohort, and validation cohort.

**Figure 3:**
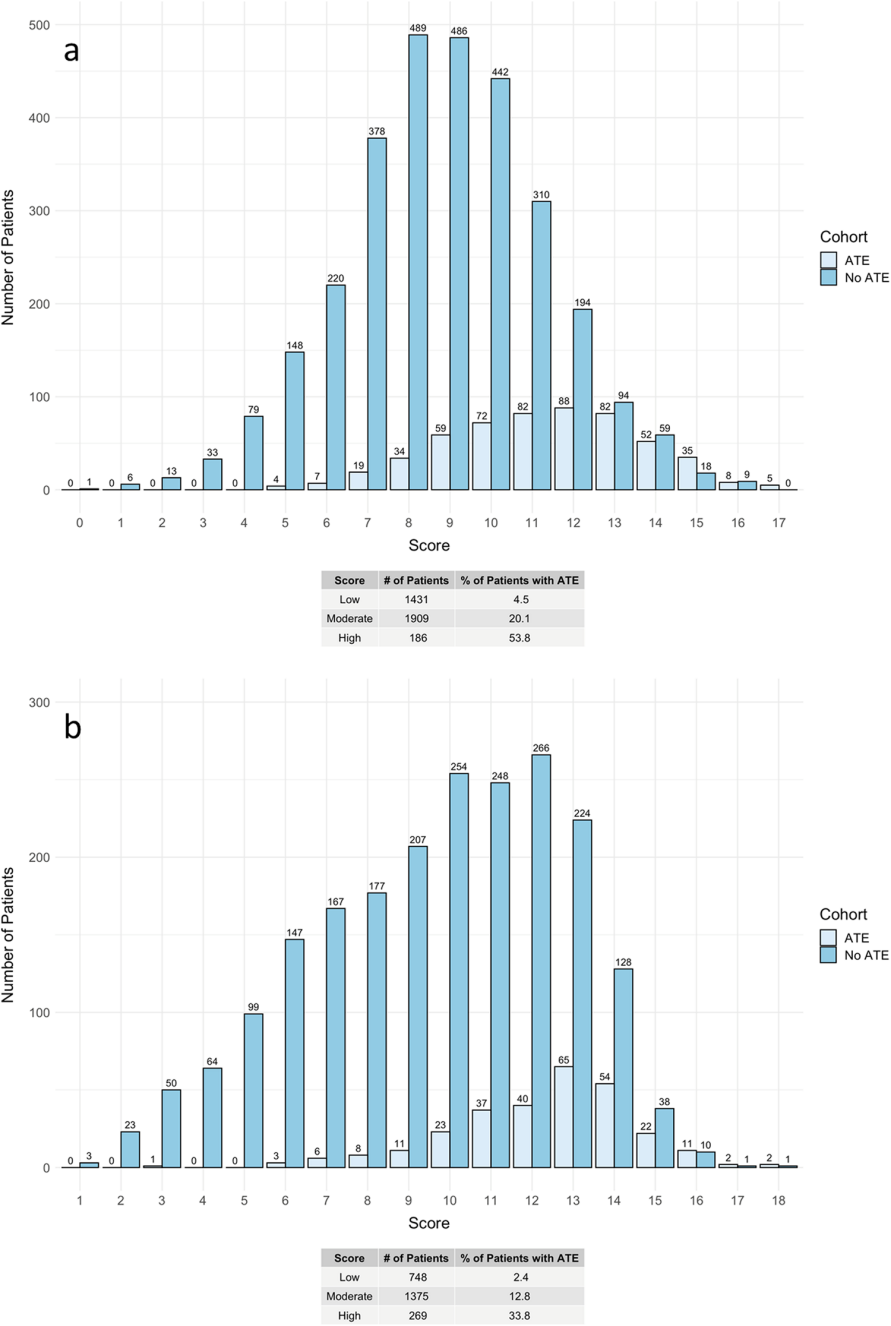
Predictive performance of the arterial thromboembolism risk score stratification in the derivation cohort (**a**) and validation cohort (**b**).

Thirty-two variables were selected from the LASSO model, then were analyzed by multivariable logistic regression with ATE as an outcome (Supplemental Table 5). A total of sixteen variables were significantly associated with ATE. Independent risk factors for ATE are presented in Fig. 1. Initial systolic blood pressure (SBP) > 160 mmHg; elevated initial biomarkers including leukocytes (> 11 K/uL), lactate dehydrogenase (> 192 U/L), interleukin-6 (IL-6) (> 5 pg/mL), troponin-I (0.04–0.09 ng/mL, > 0.09 ng/mL), B-type natriuretic peptide (BNP) (> 100 pg/mL), serum creatinine (> 1.4 mg/dL), aspartate aminotransferase (> 41 U/L); presenting hypokalemia (< 3.5 mEq/L) and hypomagnesemia (< 1.8 mg/dL); age > 60 years; male sex; and history of CVA, CAD, and cigarette smoking were associated with an increased risk of ATE (*p* < 0.05 for all comparisons). The assigned scores based on the log odds ratios (OR) of each independent risk factor are listed in Table 1, with the odds of ATE increasing by 1.6 times on average per unit increase of the score. The total ATE risk score ranges from 0 to 21 and has an AUC of 0.777 with a 95% confidence interval (CI) of 0.756–0.797 (Fig. 2) with a Brier score of 0.11. The score distribution is shown in Fig. 3 with scores stratified as low-risk (score 0–8), intermediate-risk (score 9–13), and high-risk groups (score 14 or higher), with the risk of ATE of 4.5%, 20.1%, and 53.8%, respectively. The initial letters of the weighed variables **S**ex, **A**ge, **R**ace, **S**moking and systolic blood pressure, **C**reatinine; **O**ver the normal range labs (leukocytes, lactate dehydrogenase, interleukin-6, and B-type natriuretic peptide, **V**ascular disease (cardiovascular and cerebrovascular), **A**spartate aminotransferase, **T**roponin-I, **E**lectrolyte derangement (magnesium/potassium), formed the new risk assessment model name “SARS-COV-ATE” for COVID-19–associated ATE.

**Table 1:** Scores assigned based on multivariate model.

Risk factor	Points
Male sex	1
Non-African American race	1
**Age (years)**	
40–59	2
60+	4
Presenting systolic blood pressure >=160 mmHg	1
**History**	
Cerebrovascular accident	1
Coronary artery disease	1
Smoking	1
**Labs on arrival**	
Leukocytes >11 K/uL	1
Lactate dehydrogenase >192 U/L	1
Interleukin-6 >5 pg/mL	1
Serum potassium <3.5 mEq/L	1
Serum magnesium <1.8 mg/mL	1
Troponin-I 0.04–0.09 ng/mL	1
Troponin-I >0.09 ng/mL	3
B-type natriuretic peptide >100 pg/mL	1
Serum creatinine >1.4 mg/dL	1
Aspartate aminotransferase >41 U/L	1
Maximum risk score	21

In subgroup analysis, we classified ATE events into (1) Acute coronary syndrome (unstable angina and myocardial infarction), (2) Cerebrovascular accident (acute ischemic stroke and transient ischemic attack), and (3) Other arterial thromboembolic events (intracardiac thrombus, mesenteric ischemia, and other ATE events). The risk factors for each ATE category are listed in Supplemental Tables 6 and 7.

### Risk score validation

The ATE rate was 14.8% in the bootstrapped cohort of 500 patients. The AUC was 0.711 (95% CI 0.647–0.774), and the Brier score was 0.10. Of the 2392 patients included in the validation cohort, the ATE rate was 11.9%. As shown in Supplemental Table 3B, the validation cohort tended to have more females and was younger in age compared to the training cohort. The AUC was 0.761 with a 95% CI of 0.734–0.788, and the Brier score was 0.08. Table 2 lists the sensitivity, specificity, positive predictive value, and negative predictive value of the ATE risk score stratification. The incidence of ATE was 2.4% in the low-risk group, 12.8% in the moderate-risk group, and 33.8% in the high-risk group. Compared to the low-risk group, the odds of ATE increases by 5.95 times and 20.73 times in moderate and high-risk groups, respectively.

**Table 2:** Arterial thromboembolism risk stratification and predictive performance.

	Number of patients (%)	Risk of ATE (%)	Sensitivity	Specificity	PPV	NPV	Odds ratios vs low risk
**Derivation cohort**							
Low risk (0-8)	1431 (40.6%)	4.5	–	–	–	–	–
Intermediate risk (9–13)	1909 (54.1%)	20.1	0.86	0.47	0.20	0.96	5.36
High risk (14+)	186 (5.3%)	53.8	0.61	0.94	0.54	0.96	24.84
**Validation cohort**							
Low risk (0–8)	748 (31.3%)	2.4	–	–	–	–	–
Intermediate risk (9–13)	1375 (57.5%)	12.8	0.91	0.38	0.13	0.98	5.95
High risk (14+)	269 (11.2%)	33.8	0.83	0.80	0.34	0.98	20.73

ATE, arterial thromboembolism; NPV, negative predictive value; PPV, positive predictive value.

### Discussion

Endothelial cell dysfunction induced by SARS-CoV-2 and inflammatory cytokines leading to a hyperinflammatory and hypercoagulable state is thought to be the mechanism of thromboembolism in COVID-19 infection^18^. Thrombosis is also seen in other viral infections; the interaction of platelets and influenza virus is known to induce a proinflammatory state leading to vascular occlusion^19^. Similarly, autopsies and clinical studies have shown the role of platelets in multi-organ thrombosis in COVID-19 infection^20,21^. The unfavorable prognosis associated with ATE in COVID-19 highlights the need for RAM to predict these thromboembolic complications. To our best knowledge, this is the first RAM for ATE in COVID-19 patients. We developed a model using parameters commonly available on admission. The risk of ATE was classified as low, moderate, and high based on the total score. Our RAM had a sensitivity of 0.85 and specificity of 0.81 for the high-risk group compared with the low-risk groups. The model showed a good predictive performance in the bootstrapped sample (AUC 0.711; 0.647–0.774) and the validation cohort (AUC 0.766; 0.741–0.790).

The incidence of MI is reported between 1.1 and 8.9%^2,22^ whereas stroke incidence is reported from 0.9% to 4·6% in COVID-19^2,23–25^. The incidence of ATE was 15.5% (11.9% MI and 1.3% stroke) in our derivation cohort and 13.4% (8.7% MI and 1.4% stroke) in the validation cohort. The derivation cohort included patients from March to December 2020, whereas the validation cohort included patients from January to September 2021. Despite the evolution of COVID-19 treatments, including corticosteroids, anticoagulants, antivirals, Janus kinase inhibitors, and IL-6 receptor inhibitors, the incidence of ATE did not decrease significantly over time in our cohort.

In this large, multi-institutional study, we studied composite arterial outcomes since they potentially have a similar mechanism in COVID-19 infection^18^. Moreover, there is a strong association between coronary and cerebrovascular disease^26^ which often co-occur^27^ as seen in our cohort, and one ATE event can predispose to another thrombotic complication^28^. The risk of stroke and coronary heart disease increase with age^29^. We found that age 40–59 was associated with a significantly increased risk of ATE, and the risk doubled with age ≥ 60. Moreover, we saw an increasing trend of ATE in the younger patients over time, highlighting the need for more aggressive risk factor modification in this patient group. Among sex, the risk of stroke and TIA risk are historically higher in women^30–34^. Contrary to that, the risk of MI is known to be higher in men^35^. Our study found the male sex to be an independent risk factor for ATE.

Among races and ethnicities, African-Americans have the highest risk of MI and CVA^29^. However, we found the non-African American race to be associated with a higher risk of ATE in our COVID-19 population. Racial and ethnic disparities in ATE are known^36^ and the higher prevalence of risk factors in the African-American population could contribute to an increased prevalence of ATE^37^; however, in our adjusted analysis, the African-American race had a lower incidence of ATE when provided the equal standard of care treatment. Race-specific risk factor management can potentially have positive implications in the management of COVID-19 patients. Consistent with the existing literature, history of CAD, CVA, and smoking were predictors of ATE^38–40^. Recently, SBP was shown to be the single best predictor of ATE events^41^. Systolic blood pressure ≥ 160 mmHg was a significant risk factor for ATE in our final scoring model. This underscores the need for improved blood pressure control in hospitalized COVID-19 patients. Other vital signs such as heart rate, respiratory rate, and oxygen saturation were not significant predictors in the model.

In our derivation cohort, 10.1% of patients had normal presenting troponin-I but later had ATE during hospitalization. There was a trend of high presenting troponin with the risk of ATE during hospitalization (17.5% with troponin-I 0.04–0.09 ng/ml and 34.1% patients with troponin-I > 0.09 ng/ml). Using our RAM, patients can be identified who have initial negative myocardial injury marker and do not meet the criteria for MI per the universal definition^42^, but are at risk of having MI later in the hospital course. Moreover, troponins can be elevated in CVA, could suggest a cardioembolic source^43^ and predict poor outcomes^44,45^. Among stroke patients in our cohort, 59% had troponin-I elevation, which could favor the cardioembolic etiology of stroke as atrial fibrillation is commonly seen in COVID-19^3^. However, 28.2% of patients had concomitant MI along with stroke. BNP is a biomarker of acute and chronic heart failure and acute coronary syndrome but can be seen in various other causes such as severe pneumonia, critical illness, sepsis, and metabolic insults^46^. BNP > 100 pg/mL was found to be a predictor of ATE in our model.

Leukocytosis can be reactive in severe illness; however, it is a known marker of poor outcomes in stroke and MI^47,48^ and was a risk factor in our model. Likewise, lactate dehydrogenase and IL-6 are known risk factors for cardiac and thrombotic events in COVID-19 infection^49,50.^ In terms of electrolyte imbalance, hypomagnesemia is associated with an increased incidence of hypertension, cardiovascular and cerebrovascular disease^51,52^ and predicted ATE in our patients. Hypomagnesemia may lead to secondary hypokalemia^53^ Hypokalemia is associated with an increased risk of stroke^54^ and poor outcomes in patients with MI^55^. Therefore, it is crucial to optimize electrolytes in COVID-19 patients. Creatinine elevation in COVID-19 patients can be secondary to tissue inflammation, endothelial injury, microthrombi formation, possible viral invasion, and systemic hemodynamic instability^21,56^. Furthermore, hypercreatininemia is associated with the risk of stroke and MI^57,58^, which was reflected in our RAM as well. Hepatocellular injury marker AST was found to be a predictor of ATE in our cohort. The liver injury could be the direct cytopathic effect of the virus, hepatic sinusoid thrombosis, the consequence of cytokine storm, or the aggravation of preexisting liver pathology^21,59^. Transaminitis is a possible marker of extensive myocardial injury^60^ and is known to be associated with increased mortality in MI^61^.

In our cohort, among patients who had ATE, 19.4% of patients received prophylactic anticoagulation, 54.1% received therapeutic anticoagulation, and 56.7% received antiplatelet therapy. Among patients who did not have ATE, prophylactic anticoagulation was given in 46.2%, therapeutic anticoagulation in 26.2%, and antiplatelet therapy in 27.8% of the patients (Supplemental Table 8). The effect of anticoagulation on the incidence of ATE events and the use of aspirin or a P2Y12 inhibitor as a sole antithrombotic agent is an important area that should be explored in future studies and clinical trials.

Our RAM can be used as an adjunct tool for risk stratification and assist physicians in making diagnostic choices as there is a lack of guidance for ATE prediction in COVID-19. It can also be particularly useful when diagnostic testing is not possible due to isolation precautions, limited resources, and the non-availability of staff and equipment, especially when the healthcare system is overwhelmed during the peak of a COVID-19 wave. It can help triage COVID-19 patients who present to the emergency room but are not hypoxic and might be returned otherwise. These patients may have a high risk of ATE based on our algorithm and can benefit from close monitoring. Our prognostic score provides a strong basis for further investigation and can guide selection in prospective studies and clinical trials. If validated externally in multiple independent cohorts of patients, this RAM can be implemented in daily clinical practice.

This study has both strengths and limitations. Strengths include a large, diverse population, multi-institutional nature, inclusion of critical as well as non-critical patients, and collection of data from two waves of COVID-19. The large sample size enabled us to study 49 variables and develop a powerful prediction model. Our work involves the collaborative effort of data scientists and physicians to build a robust model using careful statistical calculations. Finally, the model was validated with a marginal decrease in discrimination and preserved calibration. Limitations include the lack of time-to-event analysis and the potential for competing for risk bias. To counter this, we used the parameters which were available in the emergency room. Since our analysis includes hospitalized patients, these findings might not predict ATE in non-hospitalized patients; however, this model could be tested to evaluate the predictive performance in the outpatient setting. Moreover, we did not examine the incidence and risk of ATE beyond hospitalization. Lastly, despite the diverse nature of the Michigan population, it differs from other states in terms of racial and ethnic distribution, dietary patterns, and comorbidities^62,63^. Therefore, further studies are needed to test our model in other regions of the country.

### Conclusion

We report multiple risk factors of in-hospital ATE in a large cohort of COVID-19 patients. Our novel and robust RAM provides an accurate predictive approach for ATE in hospitalized COVID-19 patients. This risk factor-based approach can assist clinicians at the bedside in making management choices. This schema can be a useful risk-stratification tool in COVID-19 clinical care and thrombosis research.

## Supplementary Information

Supplementary Information.

## Data Availability

Data are available from the authors on request. Please contact Dr. Laila Poisson at lpoisso1@hfhs.org.
