# Supplementary material for: SARS-COV-ATE risk assessment model for arterial thromboembolism in COVID-19
Source: Sci Rep. 2022 Sep 28;12:16176. doi: 10.1038/s41598-022-18510-3 (PMC9516525; doi:10.1038/s41598-022-18510-3)
Supplement: Supplementary file 1 — Supplementary Information. [file 41598_2022_18510_MOESM1_ESM.pdf]

SUPPLEMENTAL MATERIALS

SARS-COV-ATE Risk Assessment Model for Arterial Thromboembolism in COVID-19

Pin Li, Yi Lee, Qasim Jehangir, Chun-Hui Lin, Geetha Krishnamoorthy, Anupam A. Sule, Abdul R. Halabi, Kiritkumar Patel, Laila Poisson, Girish B. Nair

Table of Contents

Page 2	Table S1. ICD-10 codes for arterial thromboembolic events
Page 3-7	Table S2. Categorization of the variables used in model building
Page 8-11	Table S3A. Comparison of patients in derivation cohort stratified by in-hospital arterial thromboembolism status
Page 12-15	Table S3B. Comparison of patients in validation cohort stratified by in-hospital arterial thromboembolism status
Page 16	Table S4. In-hospital arterial thromboembolism events in the validation and derivation cohorts
Page 17	Table S5. Variable selected by LASSO
Page 18	Table S7. Risk factors for three categories of arterial thromboembolism
Page 19	Table S7A. Multivariable analysis of risk factors for acute coronary syndrome
Page 20	Table S7B. Multivariable analysis of risk factors for cerebrovascular accident
Page 21	Table S7C. Multivariable analysis of risk factors for other arterial thromboembolic events
Page 22	Table S8A. In-hospital usage of anticoagulation and antiplatelet agents in the derivation cohort
Page 22	Table S8B. In-hospital usage of anticoagulation and antiplatelet agents in the validation cohort
Page 23	Figure S1. Consort Diagram for cohort selection of derivation cohort

Table S1. International Classification of Diseases–Tenth Revision codes for arterial thromboembolic events

**Variables**	**ICD-10 Codes**
Transient ischemic attack	G45 I65 I66
Ischemic stroke	I63
Myocardial infarction	I21, I22, I23, I24
Unstable angina	I20.0
Intracardiac thrombus	I51.3
Mesenteric ischemia	K55.0, K55.3, K55.8, K55.9
Other arterial thromboembolism	I73.9 I74 I75

Table S2. Categorization of the variables used in model building

Variable	Categorical or Continuous	Definition
Age	Categories (ref: 19-39)	19-39
		40-59
		60-79
		≥80
Body mass index	Categories (ref: 18.5-24.9)	<18.5
		18.5-24.9
		25.0-29.9
		≥30.0
Sex	Categories (ref: Male)	Male
		Female
Race/ethnicity	Categories (ref: African-American)	African-American
		Caucasian
		Hispanic
		Other
Vital Signs		
Heart rate	Categories (ref: <100)	<100: 0
		≥100: 1
Respiratory rate	Categories (ref: <20)	<20
		20-29
		≥30
Oxygen saturation	Categories (ref: Normal)	Normal: O2 Saturation >94% and no oxygen device
		Low: O2 Saturation ≤94 and any oxygen device use

Systolic blood pressure	Categories (ref: Normal)	Low: <90 mmHg
		Normal: 90-119 mmHg
		High: 120-159 mmHg
		Very High: ≥160 mmHg
**Comorbidities**	**Categories (ref: No)**	
Hypertension	Y/N	
Diabetes mellitus	Y/N	
Hyperlipidemia	Y/N	
Coronary artery disease	Y/N	
Congestive heart failure	Y/N	
Cerebrovascular accident	Y/N	Stroke and transient ischemic attack
Cancer and hematological malignancies	Y/N	Includes solid tumor and hematologic malignancies i.e., leukemia, lymphoma
Autoimmune disease	Y/N	Rheumatoid arthritis, psoriatic arthritis, spondylarthritis, systemic lupus erythematosus, Sjögren's syndrome, scleroderma, polymyalgia rheumatica, mixed connective tissue disease, dermatomyositis, polymyositis, polyarteritis nodosa, vasculitis, lupus, autoimmune hepatitis, other autoimmune disease
Liver disease	Y/N	Cirrhosis, non-alcoholic steatohepatitis, ascites
Interstitial lung disease	Y/N	Pulmonary fibrosis
Chronic obstructive pulmonary disease	Y/N	Chronic bronchitis, emphysema
Atrial fibrillation	Y/N	

Deep vein thrombosis	Y/N	
Pulmonary embolism	Y/N	
Thyroid disease	Y/N	Hypothyroidism, hyperthyroidism
Social history		
History of smoking	Y/N	
History of alcohol use	Y/N	
**Labs on presentation**		
Leukocyte (K/UL)	Categories (ref: Normal)	Low: <3.7
		Normal: ≥3.7 and ≤11
		High: >11
Lymphocytes (K/UL)	Categories (ref: Normal)	Low: <1
		Normal: 1.0-3.5
		High: >3.5
Neutrophils (K/UL)	Categories (ref: Normal)	Low: <1.5<br>Normal: 1.5 - 10<br>High: >10
Neutrophil-Lymphocyte ratio	Categories (ref: Normal)	Low: <0.8
		Normal: 0.8-3.49
		Mild: 3.5-8.99
		Moderate: 9-17.99
		Severe: >18
B-type natriuretic peptide (pg/mL)	Categories (ref: Normal)	Normal: 0-100
		High: >100
C-reactive protein (mg/dL)	Categories (ref: Normal)	Normal: 0-0.5

		High: >0.5
D-dimer (ng/mL)	Categories (ref: Normal)	Normal: 0-500
		High: >500-2000
		Very high: >2000
Ferritin (ng/mL)	Categories (ref: Normal)	Normal: ≤336
		High: >336
Lactate dehydrogenase (U/L)	Categories (ref: Normal)	Normal ≤192
		High: >192
Blood urea nitrogen (mg/dL)	Categories (ref: Normal)	Normal: ≤23
		High: >23
Creatinine (mg/dL)	Categories (ref: Normal)	Normal: ≤1.40
		High: >1.40
Total Bilirubin (mg/dL)	Categories (ref: Normal)	Normal: 0.0-1.6
		High: >1.6
Aspartate aminotransferase (U/L)	Categories (ref: Normal)	Low: <15
		Normal: 15-41
		High: >41
Alanine aminotransferase (U/L)	Categories (ref: Normal)	Low: <10
		Normal: 10-63
		High: >63
Alkaline Phosphatase (U/L)	Categories (ref: Normal)	Low: <38
		Normal: 38-126

		High: >126
Albumin (mg/dL)	Categories (ref: Normal)	Low: <3.5
		Normal: ≥3.5
Troponin-I (ng/mL)	Categories (ref: Normal)	Normal: 0-0.03 NG/ML
		High: 0.04-0.09 NG/ML
		Higher: >0.09 NG/ML
Creatine phosphokinase (U/L)	Categories (ref: Normal)	Normal: ≤280 in males or ≤155 in females
		High: >280 in males or >155 in females
Interleukin 6 (pg/mL)	Categories (ref: Normal)	Normal: 0-5
		High: >5
Lactate (mmol/L)	Categories (ref: Normal)	Normal: ≤2.2
		High: >2.2
Procalcitonin (ng/mL)	Categories (ref: Normal)	Normal: 0-0.5
		High: >0.5
Potassium (mEq/L)	Categories (ref: Normal)	Hypokalemia: <3.5
		Normal: 3.5-5
		Hyperkalemia: >5
Platelet count (K/uL)	Categories (ref: Normal)	Low: <140
		Normal: 140-440
		High: >440
Magnesium (mg/dL)	Categories (ref: Normal)	Hypomagnesemia: <1.8
		Normal: 1.8-2.5
		Hypermagnesemia: >2.5

Table S3A. Comparison of patients in derivation cohort stratified by in-hospital arterial thromboembolism status.

Variable	Total Population (N = 3526)	Arterial thromboembolism (N = 547)	No Arterial thromboembolism (N = 2979)	p-value
Sex				0.002
Male	1774 (50.3)	309 (56.5)	1465 (49.2)	
Female	1752 (49.7)	238 (43.5)	1514 (50.8)	
Age (years)				<.001
18-39	230 (6.5)	6 (1.1)	224 (7.5)	
40-59	808 (22.9)	57 (10.4)	751 (25.2)	
60-79	1598 (45.3)	261 (47.7)	1337 (44.9)	
>=80	890 (25.2)	223 (40.8)	667 (22.4)	
Race/ethnicity				0.001
Hispanic	55 (1.6)	5 (0.9)	50 (1.7)	
Other	251 (7.1)	30 (5.5)	221 (7.4)	
Caucasian	2031 (57.6)	358 (65.4)	1673 (56.2)	
African-American	1189 (33.7)	154 (28.2)	1035 (34.7)	
Body mass index (kg/m^2)				<.001
<18.5	80 (2.3)	23 (4.2)	57 (1.9)	
18.5-24.9	668 (18.9)	145 (26.5)	523 (17.6)	
25.0-29.9	1065 (30.2)	170 (31.1)	895 (30.0)	
>=30.0	1713 (48.6)	209 (38.2)	1504 (50.5)	
Heart Rate (bpm)				0.203
<100	2180 (61.8)	352 (64.4)	1828 (61.4)	
>=100	1346 (38.2)	195 (35.6)	1151 (38.6)	
Respiratory Rate (bpm)				0.003
<20	1645 (46.7)	231 (42.2)	1414 (47.5)	
20-29	1570 (44.5)	249 (45.5)	1321 (44.3)	
>=30	311 (8.8)	67 (12.2)	244 (8.2)	
Oxygen				0.009
O2Sat > 94% and no oxygen device	1524 (43.2)	208 (38.0)	1316 (44.2)	
O2Sat <=94% or any oxygen device	2002 (56.8)	339 (62.0)	1663 (55.8)	
Systolic blood pressure (mmHg)				<.001
<90	940 (26.7)	131 (23.9)	809 (27.2)	
90-119	101 (2.9)	28 (5.1)	73 (2.5)	
120-159	1978 (56.1)	281 (51.4)	1697 (57.0)	
>=160	507 (14.4)	107 (19.6)	400 (13.4)	
**Comorbidities**				
Hypertension	2734 (77.5)	455 (83.2)	2279 (76.5)	0.001
Diabetes mellitus	1446 (41.0)	234 (42.8)	1212 (40.7)	0.385
Hyperlipidemia	1677 (47.6)	284 (51.9)	1393 (46.8)	0.03
Coronary artery disease	708 (20.1)	187 (34.2)	521 (17.5)	<.001

Congestive heart failure	457 (13.0)	118 (21.6)	339 (11.4)	<.001
Cerebrovascular accident	452 (12.8)	122 (22.3)	330 (11.1)	<.001
Solid cancer and hematological malignancy	609 (17.2)	115 (21.0)	494 (16.6)	0.014
Autoimmune Disease	212 (6.0)	36 (6.6)	176 (5.9)	0.609
Liver disease*	29 (0.8)	5 (0.9)	24 (0.8)	0.796
Interstitial lung disease*	19 (0.5)	3 (0.5)	16 (0.5)	1
Atrial fibrillation	383 (10.9)	95 (17.4)	288 (9.7)	<.001
Deep vein thrombosis	286 (8.1)	56 (10.2)	230 (7.7)	0.058
Pulmonary embolism	491 (13.9)	81 (14.8)	410 (13.8)	0.561
Chronic kidney disease	491 (13.9)	101 (18.5)	390 (13.1)	0.001
End-stage renal disease	152 (4.3)	31 (5.7)	121 (4.1)	0.113
**Social history**				
Smoker	271 (7.7)	53 (9.7)	218 (7.3)	0.068
Alcohol Use	61 (1.7)	12 (2.2)	49 (1.6)	0.467
**Presenting laboratory values**				
Leukocytes				<.001
Normal	2664 (75.6)	381 (69.7)	2283 (76.6)	
Low	313 (8.9)	39 (7.1)	274 (9.2)	
High	549 (15.6)	127 (23.2)	422 (14.2)	
Lymphocytes				0.033
Normal	1375 (39.0)	207 (37.8)	1168 (39.2)	
Low	2107 (59.8)	327 (59.8)	1780 (59.8)	
High	44 (1.2)	13 (2.4)	31 (1.0)	
Neutrophils				<.001
Normal	3023 (85.7)	437 (79.9)	2586 (86.8)	
Low	77 (2.2)	8 (1.5)	69 (2.3)	
High	426 (12.1)	102 (18.6)	324 (10.9)	
Neutrophil/Lymphocyte ratio				<.001
Normal	876 (24.8)	113 (20.7)	763 (25.6)	
Low	34 (1.0)	6 (1.1)	28 (0.9)	
Mild	1561 (44.3)	216 (39.5)	1345 (45.1)	
Moderate	730 (20.7)	139 (25.4)	591 (19.8)	
Severe	325 (9.2)	73 (13.3)	252 (8.5)	
B-type natriuretic peptide				<.001
Normal	2208 (62.6)	194 (35.5)	2014 (67.6)	
High	1318 (37.4)	353 (64.5)	965 (32.4)	
C-reactive protein				0.028
Normal	81 (2.3)	5 (0.9)	76 (2.6)	
High	3445 (97.7)	542 (99.1)	2903 (97.4)	
D-dimer				<.001
Normal	1482 (42.0)	165 (30.2)	1317 (44.2)	
High	1691 (48.0)	309 (56.5)	1382 (46.4)	
Very High	353 (10.0)	73 (13.3)	280 (9.4)	
Ferritin				0.973

Normal	1501 (42.6)	232 (42.4)	1269 (42.6)	
High	2025 (57.4)	315 (57.6)	1710 (57.4)	
Lactate dehydrogenase				<.001
Normal	701 (19.9)	76 (13.9)	625 (21.0)	
High	2825 (80.1)	471 (86.1)	2354 (79.0)	
Blood urea nitrogen				<.001
Normal	2093 (59.4)	234 (42.8)	1859 (62.4)	
High	1433 (40.6)	313 (57.2)	1120 (37.6)	
Creatinine				<.001
Normal	2494 (70.7)	308 (56.3)	2186 (73.4)	
High	1032 (29.3)	239 (43.7)	793 (26.6)	
Total bilirubin				0.351
Normal	3363 (95.4)	517 (94.5)	2846 (95.5)	
High	163 (4.6)	30 (5.5)	133 (4.5)	
Aspartate transaminase				<.001
Normal	1544 (43.8)	179 (32.7)	1365 (45.8)	
Low	73 (2.1)	11 (2.0)	62 (2.1)	
High	1909 (54.1)	357 (65.3)	1552 (52.1)	
Alanine transaminase				0.036
Normal	2345 (66.5)	338 (61.8)	2007 (67.4)	
Low	307 (8.7)	52 (9.5)	255 (8.6)	
High	874 (24.8)	157 (28.7)	717 (24.1)	
Alkaline Phosphatase				0.067
Normal	3105 (88.1)	468 (85.6)	2637 (88.5)	
Low	179 (5.1)	29 (5.3)	150 (5.0)	
High	242 (6.9)	50 (9.1)	192 (6.4)	
Albumin				<.001
Normal	512 (14.5)	48 (8.8)	464 (15.6)	
Low	2347 (66.6)	438 (80.1)	1909 (64.1)	
High	667 (18.9)	61 (11.2)	606 (20.3)	
Troponin-I				<.001
Normal	2088 (59.2)	210 (38.4)	1878 (63.0)	
High	924 (26.2)	162 (29.6)	762 (25.6)	
Higher	514 (14.6)	175 (32.0)	339 (11.4)	
Creatine phosphokinase				0.031
Normal	2029 (57.5)	291 (53.2)	1738 (58.3)	
Low	303 (8.6)	44 (8.0)	259 (8.7)	
High	1194 (33.9)	212 (38.8)	982 (33.0)	
Interleukin-6				<.001
Normal	386 (10.9)	35 (6.4)	351 (11.8)	
High	3140 (89.1)	512 (93.6)	2628 (88.2)	
Lactate				<.001
Normal	2764 (78.4)	392 (71.7)	2372 (79.6)	
High	762 (21.6)	155 (28.3)	607 (20.4)	
Procalcitonin				<.001
Normal	2405 (68.2)	320 (58.5)	2085 (70.0)	

High	1121 (31.8)	227 (41.5)	894 (30.0)	
Potassium				0.009
Normal	2627 (74.5)	388 (70.9)	2239 (75.2)	
Hypokalemia	661 (18.7)	106 (19.4)	555 (18.6)	
Hyperkalemia	238 (6.7)	53 (9.7)	185 (6.2)	
Platelet Count				0.055
Normal	2788 (79.1)	416 (76.1)	2372 (79.6)	
Low	611 (17.3)	114 (20.8)	497 (16.7)	
High	127 (3.6)	17 (3.1)	110 (3.7)	
Magnesium				0.001
Normal	2591 (73.5)	369 (67.5)	2222 (74.6)	
Hypomagnesemia	750 (21.3)	135 (24.7)	615 (20.6)	
Hypermagnesemia	185 (5.2)	43 (7.9)	142 (4.8)	

*Fisher-exact tests were used for these variables; chi-square tests were used otherwise

Table S3B. Comparison of patients in external validation cohort stratified by in-hospital arterial thromboembolism status.

Variable	Total Population (N = 2392)	Arterial thromboembolism (N = 285)	No Arterial thromboembolism (N = 2107)	p-value
Sex				<.001
Male	1134 (47.4)	168 (58.9)	966 (45.8)	
Female	1258 (52.6)	117 (41.1)	1141 (54.2)	
Age (years)				<.001
18-39	358 (15.0)	14 (4.9)	353 (16.3)	
40-59	700 (29.3)	41 (14.4)	679 (31.3)	
60-79	941 (39.3)	147 (51.6)	807 (37.7)	
>=80	393 (16.4)	83 (29.1)	316 (14.7)	
Race/ethnicity				0.108
Hispanic	61 (2.6)	5 (1.8)	56 (2.7)	
Other	175 (7.3)	15 (5.3)	160 (7.6)	
Caucasian	1402 (58.6)	185 (64.9)	1217 (57.8)	
African-American	754 (31.5)	80 (28.1)	674 (32.0)	
Body mass index (kg/m^2)				0.085
<18.5	64 (2.7)	12 (4.2)	52 (2.5)	
18.5-24.9	434 (18.1)	55 (19.3)	379 (18.0)	
25.0-29.9	668 (27.9)	89 (31.2)	579 (27.5)	
>=30.0	1226 (51.3)	129 (45.3)	1097 (52.1)	
Heart Rate (bpm)				0.423
<100	1446 (60.5)	179 (62.8)	1267 (60.1)	
>=100	946 (39.5)	106 (37.2)	840 (39.9)	
Respiratory Rate (bpm)				0.001
<20	1315 (55.0)	134 (47.0)	1181 (56.1)	
20-29	962 (40.2)	127 (44.6)	835 (39.6)	
>=30	115 (4.8)	24 (8.4)	91 (4.3)	
Oxygen				0.002
O2Sat > 94% and no oxygen device	1172 (49.0)	114 (40.0)	1058 (50.2)	
O2Sat <=94% or any oxygen device	1220 (51.0)	171 (60.0)	1049 (49.8)	
Systolic blood pressure (mmHg)				<.001
<90	640 (26.8)	71 (24.9)	569 (27.0)	
90-119	51 (2.1)	11 (3.9)	40 (1.9)	
120-159	1370 (57.3)	143 (50.2)	1227 (58.2)	
>=160	331 (13.8)	60 (21.1)	271 (12.9)	
**Comorbidities**				
Hypertension	1647 (68.9)	230 (80.7)	1417 (67.3)	<.001
Diabetes mellitus	946 (39.5)	126 (44.2)	820 (38.9)	0.099
Hyperlipidemia	999 (41.8)	168 (58.9)	831 (39.4)	<.001

Coronary artery disease	373 (15.6)	103 (36.1)	270 (12.8)	<.001
Congestive heart failure	340 (14.2)	71 (24.9)	269 (12.8)	<.001
Cerebrovascular accident	293 (12.2)	62 (21.8)	231 (11.0)	<.001
Solid cancer and hematological malignancy	336 (14.0)	57 (20.0)	279 (13.2)	0.003
Autoimmune Disease	119 (5.0)	10 (3.5)	109 (5.2)	0.286
Liver disease*	38 (1.6)	4 (1.4)	34 (1.6)	1
Interstitial lung disease	301 (12.6)	45 (15.8)	256 (12.1)	0.1
Atrial fibrillation	292 (12.2)	56 (19.6)	236 (11.2)	<.001
Deep vein thrombosis	175 (7.3)	25 (8.8)	150 (7.1)	0376
Pulmonary embolism	335 (14.0)	51 (17.9)	284 (13.5)	0.054
Chronic kidney disease	319 (13.3)	69 (24.2)	250 (11.9)	<.001
End-stage renal disease	132 (5.5)	33 (11.6)	99 (4.7)	<.001
**Social history**				
Smoker	262 (11.0)	21 (7.4)	241 (11.4)	0.05
Alcohol Use	86 (3.6)	10 (3.5)	76 (3.6)	1
**Presenting laboratory values**				
Leukocytes				<.001
Normal	1802 (75.4)	184 (64.6)	1618 (76.8)	
Low	241 (10.1)	29 (10.2)	212 (10.1)	
High	349 (14.6)	72 (253)	277 (13.1)	
Lymphocytes				0.292
Normal	1093 (45.7)	119 (41.8)	974 (46.2)	
Low	1257 (52.6)	162 (56.8)	1095 (52.0)	
High	42 (1.8)	4 (1.4)	38 (1.8)	
Neutrophils				<.001
Normal	1999 (83.6)	196 (68.8)	1803 (85.6)	
Low	174 (7.3)	51 (17.9)	123 (5.8)	
High	219 (9.2)	38 (13.3)	181 (8.6)	
Neutrophil/Lymphocyte ratio				<.001
Normal	833 (34.8)	87 (30.5)	746 (35.4)	
Low	106 (4.4)	33 (11.6)	73 (3.5)	
Mild	910 (38.0)	96 (33.7)	814 (38.6)	
Moderate	352 (14.7)	34 (11.9)	318 (15.1)	
Severe	191 (8.0)	35 (12.3)	156 (7.4)	
B-type natriuretic peptide				<.001
Normal	1264 (52.8)	85 (29.8)	1179 (56.0)	
High	1128 (47.2)	200 (70.2)	928 (44.0)	
C-reactive protein				0.941
Normal	78 (3.3)	10 (3.5)	68 (3.2)	
High	2314 (96.7)	275 (96.5)	2039 (96.8)	
D-dimer				<.001
Normal	1117 (46.7)	81 (28.4)	1036 (49.2)	
High	1044 (43.6)	150 (52.6)	894 (42.4)	
Very High	231 (9.7)	54 (18.9)	177 (8.4)	

Ferritin				0.414
Normal	1040 (43.5)	117 (41.1)	923 (43.8)	
High	1352 (56.5)	168 (58.9)	11844 (56.2)	
Lactate dehydrogenase				0.003
Normal	436 (18.2)	33 (11.6)	403 (19.1)	
High	1956 (81.8)	252 (88.4)	1704 (80.9)	
Blood urea nitrogen				<.001
Normal	1620 (67.7)	129 (45.3)	1491 (70.8)	
High	772 (32.3)	156 (54.7)	616 (29.2)	
Creatinine				<.001
Normal	1852 (77.4)	177 (62.1)	1675 (79.5)	
High	540 (22.6)	108 (379)	432 (20.5)	
Total bilirubin				0.101
Normal	2254 (94.2)	262 (91.9)	1992 (94.5)	
High	138 (5.8)	23 (8.1)	115 (5.5)	
Aspartate transaminase				0.05
Normal	1333 (55.7)	141 (49.5)	1192 (56.6)	
Low	77 (3.2)	13 (4.6)	64 (3.0)	
High	982 (41.1)	131 (46.0)	851 (40.4)	
Alanine transaminase				0.423
Normal	1889 (79.0)	224 (78.6)	1665 (79.0)	
Low	146 (6.1)	22 (7.7)	124 (5.9)	
High	357 (14.9)	39 (13.7)	318 (15.1)	
Alkaline Phosphatase				0.455
Normal	2078 (86.9)	245 (86.0)	1833 (87.0)	
Low	103 (4.3)	10 (3.5)	93 (4.4)	
High	211 (8.8)	30 (10.5)	181 (8.6)	
Albumin				<.001
Normal	331 (13.8)	32 (11.2)	299 (14.2)	
Low	1482 (62.0)	214 (75.1)	1268 (60.2)	
High	579 (24.2)	39 (13.7)	540 (25.6)	
Troponin-I				<.001
Normal	588 (24.6)	24 (8.4)	564 (26.8)	
High	273 (11.4)	9 (3.2)	264 (12.5)	
Higher	1531 (64.0)	252 (88.4)	1279 (60.7)	
Creatine phosphokinase				0.005
Normal	1344 (56.2)	135 (47.4)	1209 (57.4)	
Low	218 (9.1)	28 (9.8)	190 (90)	
High	830 (34.7)	122 (42.8)	708 (33.6)	
Interleukin-6				0.001
Normal	159 (6.6)	5 (1.8)	154 (7.3)	
High	2233 (93.4)	280 (98.2)	1953 (92.7)	
Lactate				<.001
Normal	1737 (72.6)	176 (61.8)	1561 (74.1)	
High	655 (27.4)	109 (38.2)	546 (25.9)	
Procalcitonin				<.001

Normal	1407 (58.8)	118 (41.4)	1289 (61.2)	
High	985 (41.2)	167 (58.6)	818 (38.8)	
Potassium				0.001
Normal	1803 (75.4)	204 (71.6)	1599 (75.9)	
Hypokalemia	465 (19.4)	53 (18.6)	412 (19.6)	
Hyperkalemia	124 (5.2)	28 (9.8)	96 (4.6)	
Platelet Count				0.627
Normal	1906 (79.7)	228 (80.0)	1678 (79.6)	
Low	413 (17.3)	46 (16.1)	367 (17.4)	
High	73 (3.1)	11 (3.9)	62 (2.9)	
Magnesium				<.001
Normal	1527 (63.8)	114 (40.0)	1413 (67.1)	
Hypomagnesemia	443 (18.5)	38 (13.3)	405 (19.2)	
Hypermagnesemia	422 (17.6)	133 (46.7)	289 (13.7)	

*Fisher-exact tests were used for these variables; chi-square tests were used otherwise

Table S4. In-hospital arterial thromboembolism events in the validation and derivation cohorts

Events	Training cohort (n=3526)	Validation cohort (n=2392)
Transient Ischemic Attack	39 (1.1%)	24 (1.0%)
Ischemic Stroke	44 (1.3%)	34 (1.4%)
Myocardial Infarction	418 (11.9%)	207 (8.7%)
Unstable Angina	1 (0.03%)	1 (0.04%)
Intracardiac Thrombus	7 (0.2%)	6 (0.3%)
Mesenteric ischemia	2 (0.06%)	2 (0.08%)
Other Arterial Thromboembolism	88 (2.5%)	51 (2.1%)
Patients with multiple arterial thromboembolic events	51 (1.4%)*	37 (1.5%)**
**Any arterial thromboembolism**	**547 (15.5%)**	**285 (11.9%)**

*50 patients had 2 ATE events, and 1 patient had 3 ATE events.

**34 patients had 2 ATE events, 3 patients had 3 ATE events.

Table S5. Variable selected by LASSO

Gender	Atrial fibrillation	Aspartate transaminase
Age	History of deep vein thrombosis	Alanine transaminase
Race/ethnicity	Chronic kidney disease	Alkaline phosphatase
Body mass index	End-stage renal disease	Troponin-I
Heart rate	Smoker	Creatinine phosphokinase
Respiratory rate	Leukocytes	Interleukin 6
Systolic blood pressure	Neutrophil/Lymphocyte ratio	Lactate
Hyperlipidemia	B-type natriuretic peptide	Procalcitonin
Coronary artery disease	Ferritin	Potassium
Congestive heart failure	Lactate dehydrogenase	Magnesium
Cerebrovascular accident	Creatinine	

Table S6. Risk factors for three categories of arterial thromboembolism

**Acute coronary syndrome**	**Cerebrovascular accident**	**Other arterial thromboembolic events**
Age 60+ years	Respiratory rate >=30 bpm	Diabetes mellitus
Systolic blood pressure >=160 mmHg	Systolic blood pressure >=160 mmHg	History of cerebrovascular accident
Coronary artery disease	History of cerebrovascular accident	Coronary artery disease
B-type natriuretic peptide >100 pg/mL	B-type natriuretic peptide >100 pg/mL	Smoking
Aspartate aminotransferase >41 U/L	Ferritin >336 ng/mL	Leukocytes >11 K/uL
Troponin-I 0.04-0.09 ng/mL	Alanine aminotransferase >63 U/L	Magnesium <1.8 mg/dL
Troponin-I >0.09 ng/mL	Albumin >=3.9 mg/dL	
Interleukin-6 >5 pg/mL	Procalcitonin >0.5 ng/mL	
	Platelet count <140 K/UL	

Table S7A. Multivariable analysis of risk factors for acute coronary syndrome

Risk factor	Log odds	95% CI	p-value
Age 60-79	1.08	0.03 - 2.13	0.044
Age >= 80	1.30	0.22 - 2.37	0.018
Systolic blood pressure >=160 mmHg	0.42	0.01 - 0.84	0.047
Coronary artery disease	0.48	0.16 - 0.79	0.003
BNP >100 pg/mL	1.09	0.77 - 1.42	<0.001
AST >41 U/L	0.69	0.35 - 1.03	<0.001
Troponin-I 0.04-0.09 ng/mL	0.60	0.25 - 0.95	0.001
Troponin-I >0.09 ng/mL	1.90	1.55 - 2.25	<0.001
Interleukin-6 >5 pg/mL	0.92	0.18 - 1.67	0.015

Table S7B. Multivariable analysis of risk factors for cerebrovascular accident

Risk factor	Log odds	95% CI	p-value
Respiratory rate >=30 bpm	-1.29	-2.50 - -0.08	0.037
Systolic blood pressure >=160 mmHg	0.86	0.14 - 1.58	0.019
History of cerebrovascular accident	1.17	0.67 - 1.66	<0.001
B-type natriuretic peptide >100 pg/mL	0.90	0.39 - 1.41	0.001
Ferritin >336 ng/mL	-0.74	-1.23 - -0.25	0.003
Alanine aminotransferase >63 U/L	0.77	0.24 - 1.31	0.004
Albumin >=3.9 mg/dL	-0.95	-1.83 - -0.06	0.036
Procalcitonin >0.5 ng/mL	0.67	0.19 - 1.14	0.006
Platelet count <140 K/UL	-0.93	-1.7 - -0.17	0.017

Table S7C. Multivariable analysis of risk factors for other arterial thromboembolic events

Risk factor	Log odds	95% CI	p-value
Diabetes mellitus	-1.48	-2.03 - -0.93	<0.001
History of cerebrovascular accident	0.57	0.07 - 1.07	0.026
Coronary artery disease	0.70	0.24 - 1.16	0.003
Smoking	0.68	0.01 - 1.35	0.047
Leukocytes >11 K/uL	0.73	0.21 - 1.24	0.006
Magnesium <1.8 mg/dL	0.62	0.13 - 1.11	0.012

Table S8A. In-hospital usage of anticoagulation and antiplatelet agents in the derivation cohort

Drug	ATE (N: 547)	Non-ATE (N: 2979)	p-value
Prophylactic anticoagulation	480 (87.8%)	2723 (91.4%)	0.008
Therapeutic anticoagulation	124 (22.7%)	430 (11.4%)	<.001
Antiplatelet therapy	319 (58.3%)	951 (31.9%)	<.001
Aspirin	302 (55.2%)	900 (30.2%)	<.001
P2Y12 inhibitor	75 (13.7%)	163 (5.5%)	<.001
Clopidogrel	70 (12.8%)	154 (5.2%)	<.001
Prasugrel*	1 (0.2%)	3 (0.1%)	0.491
Ticagrelor*	7 (1.3%)	7 (0.2%)	0.003
Cangrelor*	1 (0.2%)	0 (0.0%)	0.155

*Fisher-exact tests were used for these variables; chi-square tests were used otherwise

Table S8B. In-hospital usage of anticoagulation and antiplatelet agents in the validation cohort

Drug	ATE (N: 285)	Non-ATE (N: 2107)	p-value
Prophylactic anticoagulation	171 (60.0%)	1483 (70.4%)	<.001
Therapeutic anticoagulation	117 (40.1%)	645 (30.6%)	<.001
Antiplatelet therapy	153 (53.7%)	464 (22.0%)	<.001
Aspirin	146 (51.2%)	445 (21.1%)	<.001
P2Y12 inhibitor	44 (13.7%)	64 (5.5%)	<.001
Clopidogrel	35 (12.8%)	62 (5.2%)	<.001
Prasugrel*	1 (0.2%)	0 (0.1%)	0.119
Ticagrelor*	12 (1.3%)	2 (0.2%)	<.001
Cangrelor*	0 (0.0%)	0 (0.0%)	1

*Fisher-exact tests were used for these variables; chi-square tests were used otherwise

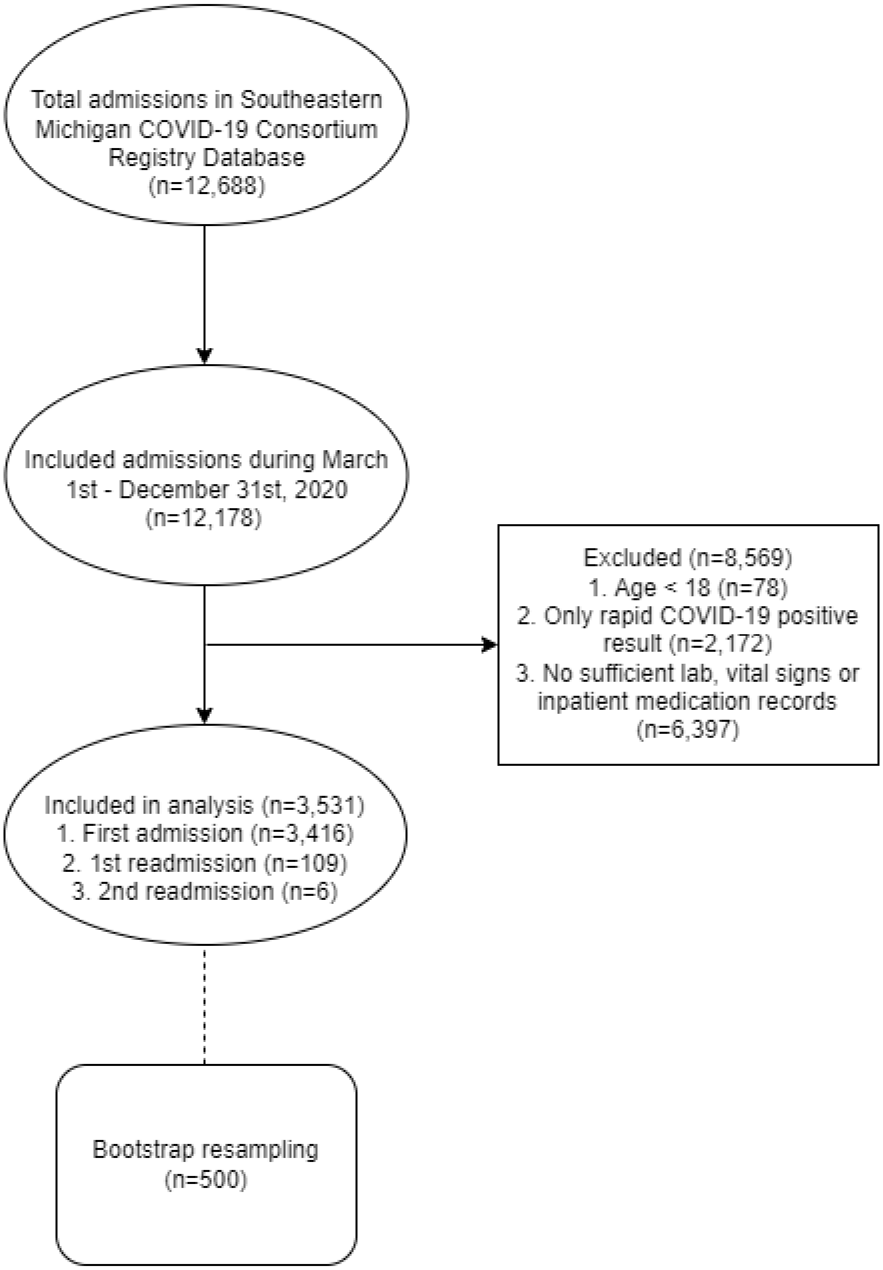

Figure S1. Consort Diagram for cohort selection of derivation cohort and the bootstrapping process.
